# Efficacy of Agmatine Treatment in Experimental Acute Pancreatitis Rat Model

**DOI:** 10.5152/tjg.2024.23017

**Published:** 2024-01-01

**Authors:** Murat Yeniçeri, Alpaslan Tanoğlu, Musa Salmanoğlu, Zafer Çırak, Mustafa Can Şenoymak, Süleyman Baş, Ayşe Sade Gökçen

**Affiliations:** 1 Department of Internal Medicine, University of Health Sciences, Sancaktepe Şehit Prof. Dr. İlhan Varank Training and Research Hospital, İstanbul, Turkey; 2 Department of Gastroenterology, Bahçeşehir University Faculty of Medicine, Göztepe Medical Park Hospital, İstanbul, Turkey; 3 Department of Internal Medicine, University of Health Sciences, Sultan Abdulhamid Han Hospital, İstanbul, Turkey; 4 Department of Internal Medicine, Ministry of Health, Honaz State Hospital, Denizli, Turkey; 5 Department of Pathology, University of Health Sciences, Sultan Abdulhamid Han Hospital, İstanbul, Turkey

**Keywords:** Acute pancreatitis, treatment, agmatine, cerulein, rat

## Abstract

**Background/Aims:**

Acute pancreatitis which is characterized by pancreatic inflammation can sometimes be difficult to treat because of limited therapeutic options. The purpose of the study was to assess the effects of agmatine in the acute pancreatitis experimental rat model.

**Materials and Methods:**

An acute pancreatitis model was created with the administration of cerulein in 40 female Sprague–Dawley rats. Agmatine was administered as a protective agent at 5 mg/kg (low dose) and 10 mg/kg (high dose). The rats were divided into 5 groups, each with 8 rats: group 1 (acute pancreatitis); group 2 (acute pancreatitis + low-dose agmatine 5 mg/kg); group 3 (acute pancreatitis + high-dose agmatine 10 mg/kg); group 4 (placebo, acute pancreatitis + saline); and group 5 (sham and saline infusion). All rats were sacrificed 24 hours after the last injection, and the levels of superoxide dismutase, interleukin-1 beta, and tumor necrosis factor-alpha were assessed in blood samples collected via cardiac puncture. Histopathological examination was performed by a pathologist, who was blind to the groups, according to the Schoenberg’s pancreatitis scoring index.

**Results:**

The amylase (16.67 and 37.89 U/L), glutathione peroxidase (13.62 and 18.44 ng/mL), tumor necrosis factor-α (39.68 and 64 ng/mL), interleukin-1 (484.73 and 561.83 pg/mL), and transforming growth factor-β (110.52 and 126.34 ng/L) levels were significantly lower and superoxide dismutase (1.29 and 0.98 ng/L) and malondialdehyde (0.99 and 0.96 nmol/mL) levels were significantly higher in group 3 compared to group 1 (*P* < .05). Moreover glutathione peroxidase, tumor necrosis factor-α, and transforming growth factor-β levels were lower, and malondialdehyde levels were higher in the group 3 compared to group 2 (*P* < .05). Although the Schoenberg’s pancreatitis scoring index was not significantly different between the high- and low-dose treatment groups, rats who received high-dose treatment had significantly lower scores compared to those with acute pancreatitis group.

**Conclusion:**

This is the first study that evaluated the efficacy of agmatine in an experimental model of acute pancreatitis. Agmatine, an anti-inflammatory and antioxidant agent, had a protective effect in an experimental rat model of acute pancreatitis.

**Main Points**

- Agmatine is an anti-inflammatory and antioxidant agent.
- Agmatine significantly decreased amylase and proinflammatory cytokine levels in experimental rat models of acute pancreatitis.
- Agmatine provided histopathological improvement in acute pancreatitis in rat models.

## Introduction

Acute pancreatitis (AP) is a gastrointestinal disorder that can sometimes be difficult to treat and can be life-threatening as a result of complications. It is a serious cause of mortality and morbidity. Generally, there is no globally accepted treatment for AP, and usually symptomatic treatment approaches are recommended. Nowadays, AP is more common in correlation with the increase in alcohol consumption and gallstones.^1^ After recurrent episodes of AP, pancreatic fibrosis leading to endocrine and exocrine hormone deficiencies may develop, which may cause gastrointestinal problems. In addition, the risk of carcinogenesis of the pancreas within 20 years in patients with chronic pancreatitis is higher than in the normal population.^1-3^ The pathophysiology of pancreatitis consists of both local destruction and systemic inflammatory response. The event that initiates the inflammation is the early activation of trypsinogen within the acinar cell.^4^ Localized tissue damage resulting from early activation of zymogens causes the secretion of the damage-associated molecular model (DAMP).^5^ The release of DAMPs causes neutrophil recruitment and initiation of inflammation.^6^ The activated neutrophils exacerbate the inflammatory process by releasing superoxide or proteolytic enzymes (cathepsin B, D, and G; collagenase and elastase). Finally, macrophages cause the release of cytokines that further mediate local (systemic in severe cases) inflammatory responses. Tumor necrosis factor-α (TNF-α), interleukin-6 (IL-6) and IL-8 are early identified mediators. These proinflammatory cytokines increase pancreatic vascular permeability, leading to bleeding, edema, and, ultimately pancreatic necrosis in severe cases.^7^

Agmatine is a nitric oxide synthase (NOS) inhibitor with anti-inflammatory and antioxidant effects. In vivo and in vitro studies have demonstrated that it inhibits fibroblast proliferation and decreases the release of inflammatory cytokines like TNF-α and IL-6.^8,9^ Agmatine acts as an inducible NOS (iNOS) inhibitor, which reduces NO overproduction, and also as an endothelial NOS (eNOS) activator with anti-inflammatory effects.^10^ Because of its biochemical and antioxidant effects, it can heal local tissue damage and systemic complications.^11^

In the current study, we evaluated the biochemical and histopathological efficacy of agmatine in a cerulein-induced AP rat model.

## Materials and Methods

This study was carried out in the Laboratory of Experimental Animals of the University of Health Sciences and was conducted with the approval of the Health Sciences University Hamidiye Animal Experiments Local Ethics Committee, dated October 16, 2019, and numbered 2019-08/04. In the study, the recommendations of the Council of Europe (European Convention for the Protection of Vertebrate Animals Used for Experimental and Other Scientific Purposes) (ETS 123) were followed.

### Experimental Model of Acute Pancreatitis

Forty female Sprague–Dawley rats weighing between 200 and 250 g were utilized. Rats were kept in a climate-controlled environment at room temperature with a 12-hour light and 12-hour dark cycle. Rats were provided free food and water intake. Food intake was discontinued 12 hours before sacrifice. Acute pancreatitis was induced by intraperitoneal (ip) injection of cerulein 40 µg/kg 4 times at 1-hour intervals.

### Study Groups

The rats were divided into 5 groups, each containing 8 animals:

**Group 1:** AP group (cerulein 40 μg/kg was administered by intraperitoneal injection 4 times at 1 hour intervals, rats were sacrificed 24 hours after the last injection).

**Group 2:** Low-dose agmatine treatment group (agmatine was administered intraperitoneally 4 times with 1-hour intervals, 5 mg/kg agmatine was administered intraperitoneally half an hour before each cerulein administration. The animals in this group were also sacrificed 24 hours after the last cerulein injection).

**Group 3:** High-dose agmatine treatment group (agmatine was administered intraperitoneally 4 times with 1-hour intervals, 10 mg/kg agmatine was administered intraperitoneally half an hour before each cerulein administration. The animals in this group were also sacrificed 24 hours after the last cerulein injection).

**Group 4:** Placebo group (AP was created as described above and 1 cm^3^ saline was administered intraperitoneally as placebo half an hour before each cerulein administration. Animals in this group were sacrificed 24 hours after the last cerulein administration).

**Group 5:** Sham group (1 mL of saline was administered by intraperitoneal injection 4 times at 1-hour intervals). The animals were sacrificed 24 hours after the last injection. No medication is given until the assessment is completed.

### Biochemical Analysis

“BT Lab rat solid phase enzyme-linked immunosorbent assay kits” was used to evaluate amylase, TNF-α, transforming growth factor (TGF)-β, malondialdehyde, glutathione peroxidase, superoxide dismutase (SOD), and IL-1 levels. Rat-specific anticores for these biochemical parameters were inserted into the spaces inside the microtiter strips. Following the addition of the biotinylated antibodies into the wells standard rat amylase, TNF-α, TGF-β, malondialdehyde, glutathione peroxidase, SOD and IL-1, unknown and control samples were pipetted. The kit instructions were followed in the measurements.

### Histopathological Evaluation

**Figure 1.**
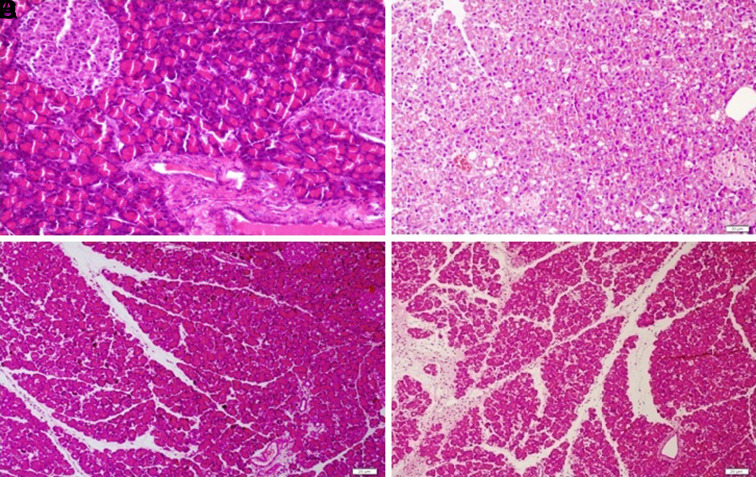
Hematoxylin and eosin staining of pancreatic tissues. (A) Normal pancreatic tissue appearance with the basophilic appearance of the cytoplasm (H&E, ×200). (B) Low-dose agmatine treatment group with mild edema and vacuolization (H&E, ×200). (C) High-dose agmatine treatment group with mild inflammation (H&E, ×200). (D) Acute pancreatitis group with inflammation, edema, vacuolization, and necrosis (H&E, ×200). H&E, hematoxylin and eosin.

Midline laparotomy was performed on rats in all groups. After blood samples were taken through cardiac puncture under anesthesia, the rats were sacrificed, and pancreatic tissues were removed for histopathological evaluation (Figure 1). Extracted tissues of the pancreas were fixed in 10% formaldehyde and then paraffinized. After hematoxylin–eosin staining, the samples were submitted for histopathological evaluation with a light microscope (Nikon Eclipse, E600) by a pathologist unaware of the groups. Inflammation, edema, vacuolization, and necrosis in pancreatic tissue samples were assessed according to the Schoenberg’s pancreatitis scoring index ranging from 0 to 4.

### Statistical Analysis

Statistical analyses were performed with Statistical Package for the Social Sciences version 20.0 software (IBM Corp.; Armonk, NY, USA). The fit of variables to normal distribution was investigated with analytical methods (Kolmogorov–Smirnov/Shapiro–Wilk tests). Results are given as mean ± SD. Comparisons of means between groups were made with the independent *t*-test or one-way analysis of variance for normally distributed data and the Mann–Whitney *U* test or the Kruskal–Wallis test for nonparametric data. *P* < .05 was accepted as statistically significant.

## Results

### Biochemical Parameters

Amylase, glutathione peroxidase (GPx), TNF-α, IL-1, malondialdehyde (MDA), TGF-β, and SOD levels were compared (Table 1). The amylase, GPx, TNF-α, IL-1, and TGF-β levels were significantly lower, and SOD, MDA levels were significantly higher in group 3 compared to group 1 (*P* < .05). Malondialdehyde and TGF-β levels did not differ between groups 2 and 4 (*P *> .05). Interleukin-1β, amylase, and TNF-α levels were significantly lower and SOD levels were significantly higher in the group 2 compared to group 4 (*P *< .05). Amylase, SOD, and IL-1β levels did not differ between groups 2 and 3 (*P* > .05). The amylase, GPx, TNF-α, IL-1, and TGF-β levels were significantly lower, and SOD and MDA levels were significantly higher in group 3 compared to group 4 (*P *< .05).

### Histopathological Score

The histopathological score differed significantly between groups (*P *< .001) (Table 2). The mean Schoenberg’s pancreatitis scoring index was the lowest in the sham group and the highest in the control group. The index did not differ between the low-dose and high-dose agmatine groups, as well as between the placebo and control groups (*P *> .05). However, the mean Schoenberg’s pancreatitis scoring index of groups 2 and 3 was significantly lower compared to both control and placebo groups (*P *< .05).

## Discussion

This is the first study to assess the therapeutic efficacy of agmatine in a rat model of AP.

There are many experimental animal models of AP. The most preferred among these is the model induced with repetitive cerulein injections, as we used in our study.^12^ In our experimental model, AP was created with subcutaneous 40 µg/kg cerulein injection 4 times at 1-h intervals, and histopathological confirmation was made. As a result of this current study, in the high-dose agmatine treatment group, the proinflammatory cytokines TNF-α, IL-1, and TGF-β levels were found to be low, and the antioxidant cytokines SOD and MDA levels were found to be high, which is a pioneer finding of positive agmatine efficacy on experimental AP model.

Currently, the exact curative treatment of AP does not exist all over the world. In recent years, many experimental studies have been carried out to find specific treatments for AP. Tanoglu et al^13^ created an experimental model of acute edematous pancreatitis in rats with repetitive injections of cerulein in their study. They used trimetazidine as a therapeutic drug, which has anti-inflammatory and anti-ischemic properties, and they described the favorable biochemical and histopathological effects of trimetazidine. Büyükberber et al,^14^ on the other hand, used caffeic acid phenethyl ester as a therapeutic agent due to its antioxidant, anti-inflammatory, and anti-proliferative properties in the experimental model of AP, and they proved its positive effects on their experimental model. In another experimental study, Özbeyli et al^15^ used astaxanthin as a therapeutic agent due to its anti-inflammatory effects in their experimental AP model. As a result of the study, they concluded that their therapeutic agents demonstrated positive results and may be a viable treatment option for pancreatitis.^15^

TGF-β is one of the important cytokines that increase in both acute and chronic pancreatitis and plays a role in the migration of inflammatory cells due to fibrosis and oxidative stress.^16^ We think that agmatine, which we used in our study, reduces complications in AP with its antioxidant and anti-inflammatory effects. It suppresses the triggering cytokines of the inflammation cascade and induces the antioxidant mechanism against oxidative stress. Bratislava et al^17^ investigated the efficacy of agmatine in the experimental model of chlorpromazine-induced hepatotoxicity. Similar to our study, they found an increase in antioxidant enzyme levels in rats given agmatine.^17^

In the study of El-Kashef et al,^18^ agmatine decreased TNF-α levels and suppressed inflammation when administered to rats with gentamicin-induced nephrotoxicity. It has also been shown that serum SOD levels and antioxidant capacity increase.^18^ Although the organs of interest were different between the studies, it is not surprising that the results were similar to our study, considering that agmatine has systemic antioxidant and anti-inflammatory effects. However, the agmatine in this study was given to the animals by oral gavage. We think that this may prevent animals from getting an equal amount of agmatine protein. In our study, agmatine was administered to each animal intraperitoneally in equal doses.

The anti-inflammatory effect of agmatine is due to its potent inhibition of cytokines such as TNF-α, IL-1, IL-2, IL-6, and interferon-gamma (IFN-γ).^19^ Tumor necrosis factor-α is a crucial cytokine in AP pathogenesis.^20^ Rashidian et al^21^ investigated the effects of agmatine on inflammatory markers in colitis and stated that agmatine reduces the levels of TNF-α and MPO levels in the serum and reduces the amount of NOS in the large intestine tissue. Agmatine also may prevent AP, as it reduces the levels of TNF-α. Therefore, our study supports the study of Rashidian et al.^21^

In conclusion, in this study, we found that agmatine, which has anti-inflammatory and antioxidant properties, has protective activity in the experimental model of AP. Agmatine may be a novel agent for the treatment of AP.

## Figures and Tables

**Table 1.** Biochemical Parameters of the Study Groups

	Group 1	Group 2	Group 3	Group 4	Group 5	*P*
Amylase (U/L)	37.89 ± 2.66	18.39 ± 0.81	16.67 ± 1.57	35.69 ± 2.38	13.36 ± 2.35	<.05^*^
Gpx (ng/mL)	18.44 ± 1.65	17.58 ± 1.33	13.62 ± 5.71	18.55 ± 1.41	14.98 ± 1.03	<.05^*^
TNF-α (ng/mL)	64.00 ± 5.03	51.96 ± 4.64	39.68 ± 4.50	63.37 ± 4.16	41.59 ± 8.35	<.05^*^
IL-1 (pg/mL)	561.83 ± 57	510.36 ± 112	484.73 ± 40	563.08 ± 60	464.44 ± 74	<.05^*^
MDA (nmol/mL)	0.96 ± 0.15	0.83 ± 0.11	0.99 ± 0.05	0.93 ± 0.11	0.70 ± 0.12	<.05^*^
TGF-β (ng/L)	126.34 ± 10.17	118.03 ± 10.72	110.52 ± 12.08	126.34 ± 10.17	101.52 ± 12.08	<.05^*^
SOD (ng/L)	0.98 ± 0.16	1.25 ± 0.12	1.29 ± 0.09	0.90 ± 0.18	1.14 ± 0.10	<.05^*^

^*^Significant differences were found between G1 and G3 in all biochemical parameters. IL-1β, SOD, amylase, and TNF-ɑ levels significantly differed between groups 2 and 4. The differences between group 3 and group 1 and also between group 3 and group 4 were statistically significant with respect to all of the biochemical parameters.

Gpx, glutathione peroxidase; IL-1, interleukin-1; MDA, malondialdehyde; SOD, superoxide dismutase; TGF-β, transforming growth factor β; TNF-α, tumor necrosis factor-α.

**Table 2.** Schoenberg Scores of the Study Groups

	Group 1	Group 2	Group 3	Group 4	Group 5	*P* ^*^
Edema	2.52 ± 0.52	1.75 ± 0.34	1.15 ± 0.14	2.55 ± 0.2	0	
Inflammation	2.12 ± 0.45	1.25 ± 0.16	1.05 ± 0.1	2.35 ± 0.16	0	
Vacuolization	1.35 ± 0.25	1.08 ± 0.4	1.03 ± 0.1	2.1 ± 0.12	0	
Necrosis	2.25 ± 0.50	0.3 ± 0.1	0.65 ± 0.1	1.13 ± 0.8	0	
Mean Schoenberg score	8.25 ± 0.71	4.38 ± 0.52	3.88 ± 0.35	8.13 ± 0.55	0	<.001

^*^The histopathological score was significantly different between the groups.

The difference between low-dose agmatine and high-dose agmatine groups and between the placebo group and the control group was not statistically significant (*P* > .05).
